# Optimising structural imaging of neuroendocrine tumours in the molecular imaging age

**DOI:** 10.1186/1470-7330-14-S1-P38

**Published:** 2014-10-09

**Authors:** Bimal Kumar Parameswaran, Kate Moodie, Michael S Hofman

**Affiliations:** 1 Peter MacCallum Cancer Centre, Melbourne, Australia

## Aim

To provide an educational update on structural imaging appearances of neuroendocrine tumours (NET), in the age of molecular imaging.

PET/CT with Ga-68 DOTA-TATE and F-18 fluorodeoxyglucose (FDG) is providing new understanding of neuroendocrine tumours including patterns and heterogeneity of disease. This is also providing new insights of structural imaging findings including CT and MRI. It is also important to be aware of the limitations of PET/CT imaging, and we outline indications where structural imaging has a high impact for patient management. These changing paradigms are translating to revised imaging protocols in our institution that are enabling personalised medicine with appropriate selection of management for an individual patient. It is also allowing us to understand the structural imaging appearances of heterogeneity within the same tumour type. A range of new targeted therapies including peptide receptor radionuclide therapy (PRRT) are now available to treat patients with non-resectable metastatic NET. New patterns of response are emerging which are important to recognise, including cystic necrosis which may initially masquerade as progressive disease due to enlargement.

We have a large population of patients with neuroendocrine tumours at Peter MacCallum Cancer Institute, providing wide experience of this spectrum of imaging findings across various subtypes of NET. We present a pictorial review of our experience.

